# Whole-Genome Sequencing of Bacterial Isolates That Degrade the Cyanobacterial Toxin Microcystin-LR

**DOI:** 10.1128/MRA.00959-20

**Published:** 2020-10-01

**Authors:** Alexander T. McCartney, Ji-Youn Yeo, Thomas M. Blomquist, Jason F. Huntley

**Affiliations:** a Bioinformatics Program, University of Toledo College of Medicine and Life Sciences, Toledo, Ohio, USA; b Department of Pathology, University of Toledo College of Medicine and Life Sciences, Toledo, Ohio, USA; c Department of Medical Microbiology and Immunology, University of Toledo College of Medicine and Life Sciences, Toledo, Ohio, USA; University of California, Riverside

## Abstract

We previously demonstrated that 13 bacterial isolates from Lake Erie, when grown in groups of four to five isolates per group, degraded the cyanobacterial toxin microcystin-LR (MC-LR) into nontoxic fragments. Whole-genome sequencing of these bacteria was performed to provide genus and species information and to predict putative MC-LR-degrading genes.

## ANNOUNCEMENT

Cyanobacteria can form large aggregations (i.e., harmful algal blooms [HABs]) that foul water bodies and threaten human health by releasing cyanotoxins, including microcystin-LR (MC-LR) (1). MC-LR causes many human health problems, including liver cancer, and its cyclic structure makes it stable in the environment (2–5). MC-LR threatens drinking water around the world, and while municipal water treatment facilities have treatment options for removing MC-LR, these processes have limited capacity, are inhibited by organic materials, are expensive, and generate other toxic by-products which must be further mitigated (2, 6, 7). Bioremediation—the use of microbes to remove contaminants or toxic materials—has been proposed as a potential solution for mitigating HAB toxins (8). For example, a *Sphingomonas* sp. was found to degrade MC-LR using the *mlrABCD* operon, and sand filters containing this bacterium removed 90% of MC-LR from contaminated water (9, 10). Subsequently, other MC-LR-degrading bacteria have been reported (11). We previously isolated and provided preliminary species identification of 13 bacterial isolates from Lake Erie that degraded MC-LR into nontoxic fragments (12). Interestingly, degradation was observed only when isolates were combined into small groups of bacteria, not by individual bacterial isolates. Additionally, *mlrABCD*-related genes were not detected in our isolates, indicating degradation by one or more unique pathways. Here, genomic sequencing was performed to better characterize these isolates and identify putative MC-LR degradation pathways.

Isolates were plated onto Reasoner’s 2A (R2A) agar medium (BD) containing 10 μg/L MC-LR (Cayman Chemical) and incubated at room temperature for 48 to 72 h, and genomic DNA was extracted from single colonies using the NucleoSpin microbial DNA kit (Macherey-Nagel). Libraries were constructed using the Nextera XT kit, and single-end sequencing was performed using the Illumina MiSeq v3 SE150 platform. The total number of reads generated for each isolate is listed in Table 1. The raw sequencing reads were filtered using BBDuk v38.79 (sourceforge.net/projects/bbmap) and Trimmomatic v0.36 (13), with default parameters. The filtered reads were assembled using SPAdes v3.13.0 (14), with a minimum contig size of 500 bp and >5× genome coverage. For 12 of the 13 isolates, genome assembly resulted in <350 contigs (Table 1). Because of high contig numbers, isolate ODNR6CL was resequenced using the Illumina v2 Micro PE150 platform and subjected to the same assembly parameters described above, resulting in 34 final contigs (Table 1). Genus (≥25% nucleotide identity) and species (≥97% nucleotide identity) designations were assigned by submitting 10-kb sequences from each isolate for NCBI BLASTn analysis (15) (Table 1). Compared to previous 16S rRNA genus and species assignments (12), genome sequencing resulted in 6 genus changes and 5 species changes (Table 1). The ranges of genera/species, genome sizes, predicted coding genes, and GC contents highlight the diversity of MC-LR-degrading bacteria (Table 1). The bacterial genomes were putatively annotated using NCBI PGAP (16). Confirming our previous results (12), no known *mlrABCD* orthologs were identified in the draft genomes. Efforts are currently focused on identifying putative MC-LR-degrading enzymes and confirming these predictions by transcriptomic analyses.

**TABLE 1:** Genomic sequencing information for 13 bacterial isolates that degrade MC-LR

Isolate name	Putative species	No. of reads generated	No. of contigs	Genome size (bp)	No. of coding genes	Genome coverage (×)	GC content (%)	*N*_50_ (bp)	SRA accession no.	GenBank accession no.
ODNR4P	*Emticicia* sp.^*a*^	2,388,911	225	6,616,476	5,087	54.16	37.5	80,332	SRX7288241	JAAAKT010000000
ODNR4SY	Pseudomonas putida^*b*^	841,168	165	5,754,181	5,121	21.93	62.0	96,929	SRX7288242	JAAAKU010000000
CRIBPO	*Emticicia* sp.^*a*^	3,179,355	39	6,060,560	5,119	78.69	43.7	416,347	SRX7288246	JAAAKV010000000
SLFW	*Pseudomonas* sp.^*b*^	996,033	55	6,493,286	5,774	23.01	60.2	317,538	SRX7288247	JAAAKW010000000
ODNR1LW	*Pseudomonas* sp.^*b*^	1,244,030	343	9,409,464	8,628	19.83	62.2	63,532	SRX7288248	JAAAKX010000000
ODNR6CL	Pseudomonas monteilii^*a*^	1,031,645	34	5,682,232	5,135	27.23	62.2	857,913	SRX7288249	JAAAKY010000000
CRIBMP	*Runella* sp.^*b*^	2,113,625	57	7,136,464	5,783	44.43	44.8	400,856	SRX7288250	JAAAKZ010000000
SLTY	*Caulobacter* sp.^*a*^	1,536,802	87	4,082,691	3,988	56.46	68.3	103,333	SRX7288251	JAAALA010000000
BC115SP	*Cellulophaga* sp.^*a*^	2,804,750	208	7,086,307	5,482	59.37	37.2	130,583	SRX7288252	JAAALB010000000
1163BD	Sphingobium yanoikuyae	1,006,399	148	5,459,634	5,014	27.65	64.4	96,382	SRX7288253	JAAALC010000000
CRIBSB	*Rhizobium* sp.^*a*^	1,247,980	180	7,187,913	6,853	26.04	63.0	124,346	SRX7288243	JAAALD010000000
SLTP	*Porphyrobacter* sp.	957,166	35	3,409,488	3,164	42.11	64.6	178,339	SRX7288244	JAAALE010000000
BC115LW	*Pseudomonas* sp.^*b*^	731,624	124	5,904,409	5,315	18.59	61.0	75,282	SRX7288245	JAAALF010000000

a Indicates new genus and species assignments, compared to the previous publication (12).

b Indicates revised species assignment, compared to the previous publication (12).

### Data availability.

All sequencing data are available in the NCBI Sequence Read Archive (SRA), and the assembled genomes are available in GenBank (see Table 1 for accession numbers). The collective data are available under BioProject accession number PRJNA593853.
